# Higher expression of circulating miR-182 as a novel biomarker for breast cancer

**DOI:** 10.3892/ol.2013.1593

**Published:** 2013-09-24

**Authors:** PING-YU WANG, HAI-TAO GONG, BAO-FENG LI, CHUN-LEI LV, HUAN-TAI WANG, HUI-HUI ZHOU, XIN-XIN LI, SHU-YANG XIE, BAO-FA JIANG

**Affiliations:** 1 School of Public Health, Shandong University, Jinan, Shandong 250012, P.R. China; 2 Key Laboratory of Tumour Molecular Biology in Binzhou Medical University, Department of Biochemistry and Molecular Biology, Binzhou Medical University, Yantai, Shandong 264003, P.R. China; 3 Laiyang Central Hospital of Weifang Medical College, Yantai, Shandong 264000, P.R. China; 4 Shandong China Traditional Medical Affiliated Hospital, Jinan, Shandong 250012, P.R. China; 5 Department of Pathology, Affiliated Yuhuangding Hospital, Medical College of Qingdao University, Yantai, Shandong 264000, P.R. China

**Keywords:** miR-182, circulating miRNAs, breast cancer, diagnosis, gene expression

## Abstract

MicroRNAs (miRNAs), present in the serum in a stable and reproducible manner, may be used as biomarkers for various diseases. Few studies have previously investigated circulating miRNAs in the peripheral blood of breast cancer (BC) patients. To identify the role of serum miR-182 levels in BC, the present study detected miR-182 levels in the serum of 46 BC patients and 58 controls, by quantitative PCR. The results showed that the serum miR-182 levels in BC patients were significantly higher compared with the serum of healthy controls (P<0.01). The miR-182 was also overexpressed in the BC tissues compared with the para-carcinoma tissues. Furthermore, the serum levels of miR-182 in the estrogen receptor (ER)-positive patients were considerably lower compared with those in the ER-negative patients. The serum levels of miR-182 in the progesterone receptor (PR)-positive patients were also found to be lower compared with those in the PR-negative patients. The current study highlights results consistent with miR-182 as a novel and valuable biomarker for the diagnosis of BC.

## Introduction

Breast cancer (BC) is the most common type of cancer in females worldwide. BC originates most commonly from the inner lining of milk ducts or lobules of breast tissue (1), which accounts for 22.9% of all types of cancer (with the exception of non-melanoma skin cancer) in females and has caused 458,503 mortalities worldwide (13.7% of cancer mortality in females) in 2008. According to the American Cancer Society, almost 230,000 new cases and 40,000 mortalities occurred in the United States in 2011. However, recognized risk factors of BC may be absent in 50–80% of patients (2), which establishes an increased interest to identify possible risk factors that contribute to BC.

microRNAs (miRNAs) are small (~22 nucleotides), non-coding RNA molecules. miRNAs, which modulate the expression of targeting genes by post-transcription, are involved in the regulation of various cell processes, including apoptosis, hematopoietic cell differentiation, metabolism, neural development and metastasis (3,4). A number of miRNAs are involved in several types of human cancer, including BC. The majority of previous studies have described the profile of miRNA expression in BC cell lines and primary tumor tissues. For example, increased expression of the miR-191/425 cluster in aggressive BC cells changes global gene expression profiles, which has a fundamental impact on the progression of BC cells (5). By analyzing the miR-21 expression in BC tissues, Ozgün *et al* previously reported that patients with high miR-21 expression levels have a significantly lower disease-free survival than patients with low miR-21 expression levels, which indicates that miR-21 is an indicator of an aggressive BC phenotype (6). The overexpression of miR-21 increases BC MCF-7 cell growth, migration and invasion, self-renewal and clonogenicity (7). The overexpression of miR-200a protects tumor cells from anoikis and promotes metastases, while inhibition of miR-200a suppresses anoikis resistance in BC cells (8). The decreased miR-200f expression is likely to increase the expression levels of EMT-transcriptional inducers and may be used as a hypothetical biomarker to assess the occurrence of EMT in BC (9). Let-7, as a tumor suppressor, inhibits the estrogen receptor (ER) α-mediated cellular malignant growth in ER-positive BC stem cells (10). The abovementioned studies show that miRNAs are important for the tumorigenesis, migration and invasion of BC.

The apoptotic and necrotic primary tumor discharges miRNAs into the blood circulation, known as circulating miRNAs. Therefore, blood contains circulating miRNAs from numerous cells (including tumor cells), which makes it possible to detect miRNAs from specific organs, tissues or cells using surface markers for proper quantification (11,12). Moreover, the circulating miRNAs, resistant to RNase activity, are rare and extremely stable in serum and plasma (13). This stability translates into consistent miRNA expression levels among individuals, which makes serum miRNAs attractive biomarkers for the diagnosis of BCs. However, there have been only a few previous publications investigating circulating miRNAs in the peripheral blood of BC patients (13–16). The present study investigated the levels of miR-182 in the blood serum of BC patients to identify the potential of serum miRNAs as biomarkers for BC.

## Materials and methods

### Subjects

The present study was performed at the Inpatient Department of Medical Oncology of Laiyang Central Hospital (Yantai, China). The research protocol was approved by the Medical Ethics Committee of Binzhou Medical University (Yantai, China). All experiments were performed according to the relevant guidelines of the Medical Ethics Committee of Binzhou Medical University.

In total, 46 BC patients, aged 30–79 years, were pathologically diagnosed with BC, for the first time, between May 1st, 2010 and September 30th, 2012. The patients had not received prior chemotherapy. Healthy controls (n=58), came to Laiyang Central Hospital for physical examination between May 1st, 2010 and September 30th, 2012 and were diagnosed without any tumor or physical illness. Prior to inclusion, all the eligible BC patients and healthy controls provided written informed consent following a full explanation of the study procedures.

### Immunohistochemistry

Histological sections (3-μm) were deparaffinized in xylene and rehydrated. Antigen retrieval was performed by microwaving the sections in 10 mM citric acid monohydrate. Endogenous peroxidase activity was blocked by 0.5% H_2_O_2_ treatment. The slides were incubated with appropriate dilutions of the primary antibodies [anti-ER, 1:200; and anti-progesterone receptor (PR), 1:200; ZSGB-BIO, Beijing, China] at 4°C overnight. The same procedure was performed for negative controls which were incubated overnight in 1X PBS without antibody. The reaction was visualized by the ABC Kit (ZSGB-BIO) and positive ER and PR status was defined by nuclear staining of >10%.

### miRNA isolation from serum and tissue

Serum samples from the patients and controls were collected between 7:00 and 8:00 a.m. Following centrifugation for 30 min at 2,650 g, plasma samples were stored at 80°C. miRNAs were extracted from plasma by the mirVana™ miRNA isolation kit (Ambion, Carlsbad, CA, USA) according to the manufacturer's instructions. Tissue samples were homogenized in a denaturing lysis solution. Total RNA was extracted from tissue lysis using the TRIzol reagent (Invitrogen Life Technologies, Carlsbad, CA, USA). Then, miRNA was separated from 30–50 mg of total RNA using the Ambion miRNA Isolation Kit (Ambion).

### Quantitative PCR (qPCR)

miRNAs were added poly (A) tails by poly (A) polymerase (Ambion). The cDNAs were synthesized by a real-time primer, 5′-AACATGTACAGTCCATGGATGd(T)30N(A,G,C or T)-3′. miR-182 was then analyzed by qPCR and the primer used was: forward, 5′-GGCAATGGTAGAACTCACACT-3′ and reverse, 5′-AACATGTACAGTCCATGGATG-3′. qPCR analysis was performed using SuperTaq Polymerase (Takara Biotechnology Co., Ltd., Dalian, China). miR-182 expression was detected using the RG3000 system (Corbett Life Science, Mortlake, Australia) with the Quantitect SYBR-Green Kit (Qiagen, Hilden, Germany) as follows: initial denaturation at 95°C for 5 min, followed by 40 cycles of 95°C denaturation for 20 sec, 52°C annealing for 20 sec and extension at 72°C for 30 sec. Fluorescence was observed at 585 nm at each extension step of 72°C. Human 5S rRNA was added into each sample and served as a control. All experiments were repeated in triplicate.

### Statistical analysis

Data were first tested for normal distribution and variance homogeneity using the Shapiro-Wilk test and F-test, respectively. Data are presented as mean ± SD for normal distributions, otherwise, data are presented as median and quartiles. Since age, height and weight showed normal distributions, differences between these groups were analyzed using the Student's t-test. However, when the levels of miR-182 did not show a normal distribution, non-parametric tests were applied. miR-182 continuous variables between groups were analyzed by the Wilcoxon rank-sum test. Statistical analyses were performed using R version 2.15.0^©^ (2012; ISBN 3-900051-07-0). P<0.05 was considered to indicate a statistically significant difference.

## Results

### Clinical characteristics of patients

In total, 46 BC patients and 58 controls participated in the present study. The demographic and clinical characteristics of all the patients and controls are provided in Table I. No differences in age, height and weight were found between the BC patients and their controls. Of the 46 patients, 29 patients were ER-positive (63.0%) and 28 PR-positive (60.9%) in the entire tumor set (46 cases). Alcohol and passive smoking have been reported to increase BC risk (17–19), but no significant differences were identified between the BC patients and their controls in the present study (Table I).

### Higher expression of miR-182 in BC tissues

**Figure 1:**
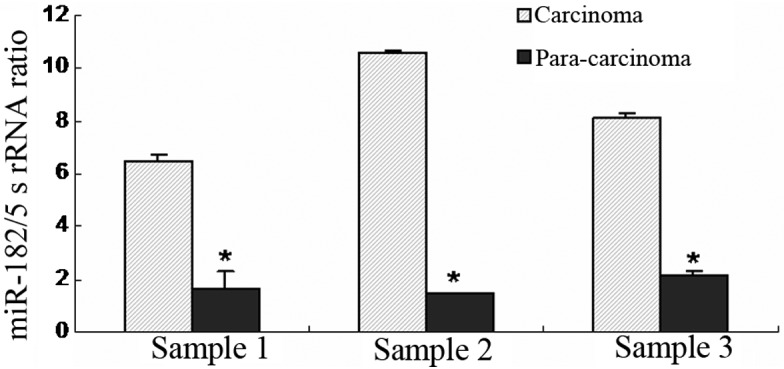
miR-182 expression in BC tissues. qPCR results showed that miR-182 expression was considerably higher in the BC tissues compared with control tissues (P<0.01). Samples 1–3; three BC and para-carcinoma tissues. 5S rRNA was used as a control. BC, breast cancer; qPCR, quantitative PCR.

miR-182, as an oncogene, is important for the development of BC (20,21). To further demonstrate the role of miR-182 in BC, its expression was detected in the BC tissues. The results showed that miR-182 expression was markedly increased (>4-fold higher) in BC tissues (n=3) compared with paracancerous tissues (n=3) (Fig. 1), which is consistent with the oncogenic role of miR-182.

### Higher levels of miR-182 in the serum of patients with BC

**Figure 2:**
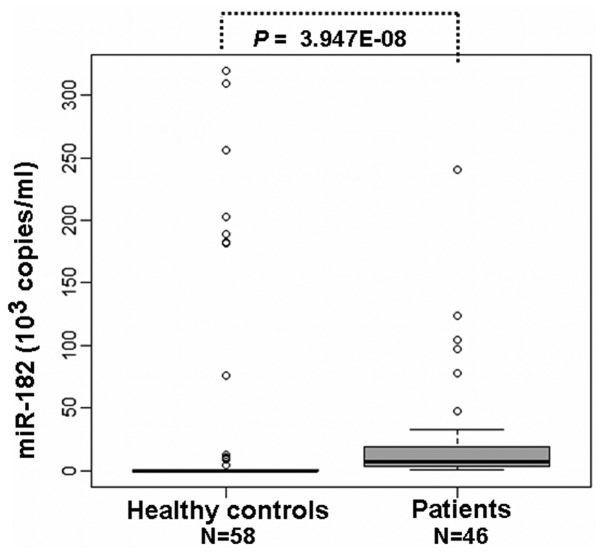
Serum miR-182 levels in BC patients and their controls. qPCR showed that serum miR-182 levels in BC patients (n=46) were considerably higher compared with healthy controls (n=58) (P=3.947E^−08^). BC, breast cancer; qPCR, quantitative PCR.

Furthermore, the serum levels of miR-182 were detected by qPCR to investigate the role of miR-182 in the diagnosis of BC. It was found that the serum miR-182 levels in BC patients were 7.075×10^3^ copies/ml (n=46), which were significantly higher compared with the serum of healthy controls (0.003×10^3^ copies/ml) (P<0.01; n=58; Table I; Fig. 2). The results demonstrated that the serum levels of miR-182 were higher in BC, indicating that miR-182 may also be an important factor for the pathogenesis of BC.

### Correlation of ER/PR with circulating miR-182 in the serum of BC patients

**Figure 3:**
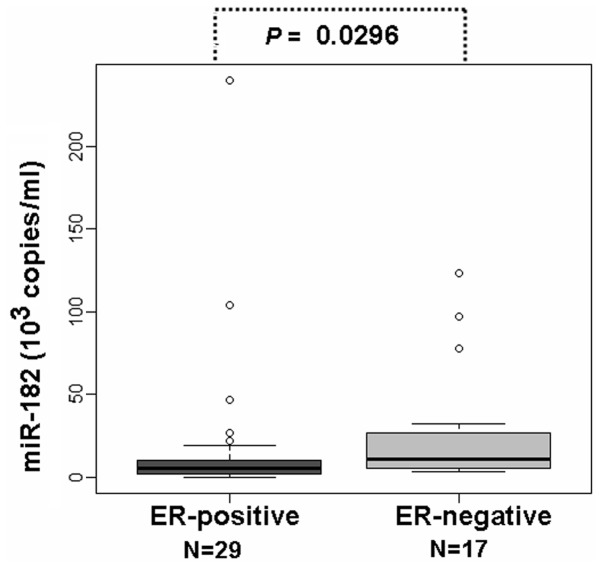
Correlation between serum miR-182 levels and ER-positive expression in BC patients. qPCR showed that serum miR-182 levels were considerably lower in ER-positive BC patients compared with ER-negative BC patients (P=0.0296). ER, estrogen receptor; BC, breast cancer; qPCR, quantitative PCR.

**Figure 4:**
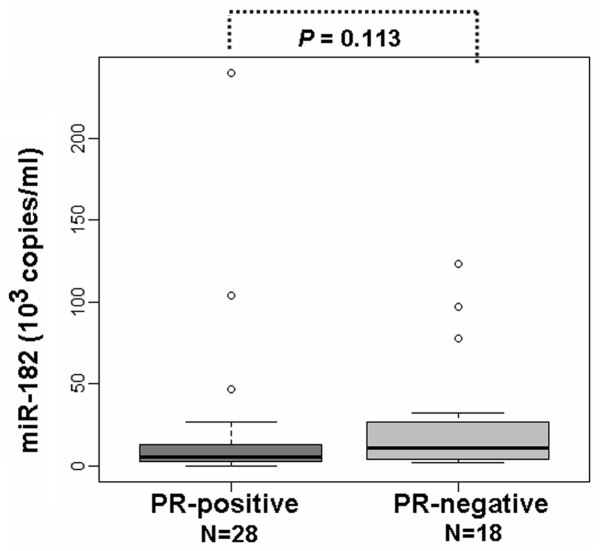
Correlation between serum miR-182 levels and PR-positive expression in BC patients. qPCR showed that serum miR-182 levels were lower in PR-positive BC patients compared with ER-negative BC patients, but there was no statistically significant difference. PR, progesterone receptor; BC, breast cancer; ER, estrogen receptor; qPCR, quantitative PCR.

ER and PR are important factors associated with the etiology and therapy of BC (22,23). To study the correlation between ER and PR with the serum levels of miR-182, the serum levels of miR-182 were detected in ER- and PR-positive patients and compared with ER- and PR-negative patients. The results showed that the serum levels of miR-182 in the ER-positive patients (n=29) were 5.41×10^3^ copies/ml, considerably lower compared with the ER-negative patients (n=17) (P<0.05; Table II; Fig. 3). The serum levels of miR-182 in the PR-positive patients (n=28) were also found to be lower compared with the PR-negative patients (n=18) (Table II; Fig. 4).

## Discussion

The ideal biomarkers for BC diagnosis should be easily accessible in order that they may be sampled relatively non-invasively. In addition, biomarkers must be sensitive enough to be detected in early stage tumors in almost all patients, while absent or minimal in healthy control individuals (24). miRNAs are markedly stable molecules, preserved well in formalin-fixed and fresh snap frozen specimens (25, 26). Their expression profiles are pathognomonic or tissue-specific in tumors (27), which establishes them as an ideal class of biomarker for BC diagnosis. In the current study, miR-182 was isolated from the tissues of BC patients and healthy controls and it was found that miR-182 expression was considerably higher in the BC tissues compared with the control tissues. This result is consistent with the oncogenic role of miR-182 in various types of cancer. Furthermore, miR-182 was isolated from the serum of BC patients and controls. The results demonstrated that miR-182 levels in the serum of BC patients were also markedly higher than those of the controls, indicating miR-182 is a useful biomarker for BC diagnosis.

Previous miRNA expression studies in BC have indicated the importance and potential roles of miRNA as disease classifiers and prognostic tools. Iorio *et al*(28) previously identified that 29 miRNAs were differentially expressed in BC tissues compared with control tissues. In addition, Mattie *et al* reported unique sets of miRNAs associated with BCs, which were defined by their roles of HER2/neu or ER/PR status (29). It has been previously reported that the pre-miR-27a rs895819 polymorphism may be associated with BC susceptibility or cancer development in Caucasians (30). The C allele of hsa-miR-146a (31) and hsa-miR-196a2 rs11614913 SNP (32), associated with BC risk, were also demonstrated to be important in familial breast/ovarian tumor development. These studies indicated that miRNAs are crucial in the development and diagnosis of BC.

Recently, several studies support that miR-182 acts as an oncogene in the development of BC (20,21,33,34). miR-182 is overexpressed in human BC tissues and cell lines (MB-231 cells) and β-catenin binds to the promoter to increase the expression of miR-182 (20). Highly expressed miR-182 functions as a potential oncomir in BC (21), which disrupts the homologous recombination pathway in BC tissues. Mechanistically, the overexpression of miR-182 decreases BRCA1 protein levels and impedes DNA repair, while antagonizing miR-182 enhances BRCA1 levels and induces resistance to the poly (ADP-ribose) polymerase 1 inhibitor (33). FOXO1, a putative tumor suppressor, is also a target of miR-182 and an antisense inhibitor specific to miR-182 which leads to a significant increase in endogenous FOXO1 expression (34). Similarly, the current study demonstrated that miR-182 was upregulated in BC tissues compared with control tissues, consistent with the important role of miR-182 in the tumorigenesis of BC.

miRNAs have been previously demonstrated to be present in the serum in a stable and reproducible manner. In addition, the unique expression patterns of serum miRNAs may be used as biomarkers for various diseases (13,35). Using qPCR, miR-205 was demonstrated to be downregulated, while miR-155 was upregulated in BC patient serum (36). The plasma levels of circulating miR-10b and miR-373 were found to be significantly higher in BC patients with lymph node metastasis compared with normal controls (37). In contrast to increased miR-21 levels, circulating miR-92a levels were decreased in the BC patients compared with the controls (38). Although miR-182 has been reported to be important for BC tumorigenesis, no previous studies have analyzed the role of circulating miR-182 in the diagnosis of BC. To explore the diagnostic role of circulating miR-182 in BC, the present study isolated miRNAs from the serum of BC patients and healthy control individuals. The results revealed that the miR-182 levels in BC were higher compared with the healthy controls, which confirmed the diagnostic role of miR-182 in BC.

The prognostic and therapeutic roles of ER or PR in BC have been studied extensively and are well established (22,23,39). Significant associations have been found between ER- and PR-positive rates with menopausal status, tumor size or the presence of distant metastases in BCs (40). In total, >75% of primary BC patients express ER and ~50% of these tumors are stained positively with PR4. The results of the present study also showed that 64.4% of primary BC patients express ER and 62.2% of these patients PR. The effects of ER- and PR-positive expression on the serum levels of miR-182 were further investigated and it was found that serum levels of miR-182 were lower in the ER- and PR-positive patients compared with the ER- and PR-negative subjects. The results indicated that there is a close correlation between serum levels of miR-182 and ER- and PR-positive expression in BC patients. Although alcohol and passive smoking increases BC risk (17–19), in the present study, no significant differences were identified between the BC patients and their controls. The relatively small number of available previous studies may lead to this limitation.

In summary, the results of the present study showed that the levels of miR-182 in the serum of BC patients were upregulated compared with healthy controls. Notably, the levels of miR-182 in the serum of ER-positive patients was considerably lower compared with the ER-negative patients. Overall, the present study highlights miR-182 as a novel diagnostic marker for BC.

## Figures and Tables

**Table I:** Demographic and clinical characteristics of the study samples.

Characteristics	Healthy controls	Patients	P-value^a^
n	58	46	
Age (mean ± SD), years	52.00±9.81	48.30±10.03	0.060
Weight (mean ± SD), kg	65.31±8.63	66.00±8.65	0.400
Height (mean ± SD), cm	157.98±4.63	158.76±4.64	0.680
ER-positive/negative, n	-	29/17	-
PR-positive/negative, n	-	28/18	-
Non-alcoholic/alcoholic drinks, n	57/1	41/5	0.085
Non-passive/passive smokers, n	27/31	14/32	0.095
Median miR-182, n	0.003^b^	7.075^b^	3.947E^−08^^b^

a P-values were obtained from the Student's t-test or Wilcoxon rank-sum test, comparing patient samples with control samples;

b ×10^3^ copies/ml.

ER, estrogen receptor; PR, progesterone receptor.

**Table II:** Correlation between ER- and PR-positive samples with miR-182.

Receptor	n	Median miR-182	P-value^a^
ER			
Positive	29	5.409^b^	0.0296
Negative	17	10.648^b^	
PR			
Positive	28	5.395^b^	0.1130
Negative	18	10.643^b^	

a P-values were obtained from the Wilcoxon rank-sum test, comparing ER- and PR-positive samples with ER- and PR-negative samples;

b ×10^3^ copies/ml.

ER, estrogen receptor; PR, progesterone receptor.
